# Bridging piezoelectric and electrostatic effects: a novel piezo-MEMS pitch/roll gyroscope with sub 10°/h bias instability

**DOI:** 10.1038/s41378-024-00773-7

**Published:** 2024-10-30

**Authors:** Zhenxiang Qi, Bowen Wang, Zhaoyang Zhai, Zheng Wang, Xingyin Xiong, Wuhao Yang, Xiaorui Bie, Yao Wang, Xudong Zou

**Affiliations:** 1 The State Key Laboratory of Transducer Technology, Aerospace Information Research Institute, Chinese Academy of Sciences, Beijing, China; 2 School of Electronic, Electrical and Communication Engineering, University of Chinese Academy of Sciences, Beijing, China; 3 Qi Lu Aerospace Information Research Institute, Jinan, China

**Keywords:** Sensors, Engineering

## Abstract

This paper proposes a novel piezo-MEMS pitch/roll gyroscope that co-integrates piezoelectric and electrostatic effects, for the first time achieves electrostatic mode-matching operation for piezoelectric gyroscopes. Movement of operated out-of-plane (OOP) mode (*n* = 3) and in-plane (IP) mode (*n* = 2) are orthogonal, ensuring that the OOP amplitude is not significantly limited by parallel plates set at nodes of IP mode. Therefore, a large OOP driving amplitude actuated by piezoelectric and frequency tuning in the IP sense mode trimmed by electrostatic can be achieved together with a low risk of pull-in, hence releases the trade-off between the tuning range and the linear actuation range. At a tuning voltage of 66 V, the frequency split decreased from 171 Hz to 0.1 Hz, resulting in a 167x times improvement in sensitivity. The mode-matched gyroscope exhibits an angle random walk (ARW) of 0.41°/√h and a bias instability (BI) of 8.85°/h on a test board within a customized vacuum chamber, marking enhancements of 68x and 301x, respectively, compared to its performance under mode-mismatch conditions. The BI performance of the presented pitch/roll gyroscope is comparable to that of the highest-performing mechanically trimmed piezo-MEMS yaw gyroscopes known to date, while offering the unique advantage of lower cost, better mode-matching resolution, and the flexibility of real-time frequency control.

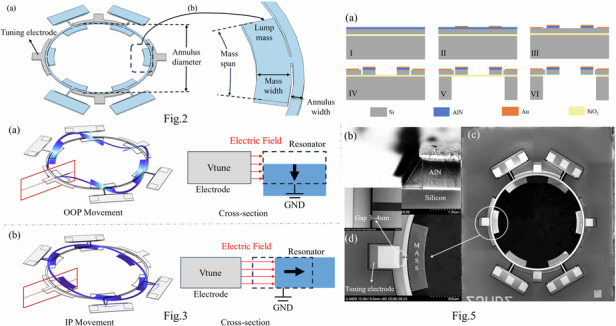

## Introduction

MEMS gyroscopes, which play an important role in inertial navigation systems, have received widespread research and attention due to their small size and light weight^1^. The electrostatic mode-matched gyroscope effectively improves the signal-to-noise ratio (SNR) by Q-amplification of the rate response^2,3^. However, the capacitive gaps limit the linear actuation range^4^ and hinder the process of high-performance gyroscopes^5^.

Piezoelectric gyroscopes have the characteristics of high electromechanical coupling efficiency and a large linear actuation range, presenting a viable avenue to break the performance barriers faced by MEMS gyroscopes^6^. However, due to the lack of effective tuning mechanisms, achieving mode matching operation and cross-mode decoupling on piezoelectric resonators is challenging. This has resulted in the Bias Instability (BI) performance of piezoelectric gyroscopes not being on par with that of state-of-the-art electrostatic mode-matched gyroscopes^7–10^.

Leveraging the frequency control research conducted on piezoelectric resonators may offer a pathway to identify suitable mechanisms for application in piezoelectric gyroscopes. Piezoelectric stiffening or the ferroelectric effect can be utilized for relative frequency and eigenmode control of a piezoelectric resonator^11–13^, but the complex electrode layout for multi-mode resonators makes it challenging to apply them to Thin-Film Piezoelectric-on Substrate(TPoS) gyroscopes. Mechanical trimming, which involves depositing or removing mass on a resonator using a laser or focused ion beam, is a possible frequency control approach. Piezoelectric gyroscopes can achieve tactical-level performance using laser trimming^14^. Nevertheless, precise tuning requires modeling the vibration mode of the gyroscope and multiple iterations^15,16^ which increases the overall cost. Moreover, the fixed frequency after trimming is not suitable for real-time frequency control situations, thereby reducing the long-term stability of the gyroscope^17,18^.

Co-integrating electrostatic effects with piezoelectricity holds promise for improving the mode-matching resolution and noise performance of piezoelectric gyroscopes, and, importantly, as the tuning is electrical, it does not incur additional costs on device. Previous research has successfully applied electrostatic tuning to aluminum nitride on silicon(AlN-on-Si) free-free beam resonators and accelerometers^19,20^. However, implementing such a tuning mechanism within piezoelectric gyroscopes has received relatively little research, mainly due to the trade-off between the driving mode amplitude and the tuning range^18,21^. Pull-in occurs when the displacement of parallel plates exceeds 1/3 of the gap width, significantly compromising the benefit of a large linear actuation range of piezoelectric gyroscopes.

In this paper, we co-integrate piezoelectric and electrostatic effects in a AlN-on-Si novel pitch or roll gyroscope, achieving electrostatic mode-matching operation for piezoelectric gyroscopes for the first time. The electrostatic parallel plate electrodes of the gyroscope are only set in the IP direction. Due to the absence of parallel plates in the OOP direction, the OOP mode is selected as the driving mode to achieve a large driving displacement. The IP mode serves as a sensing mode, and its eigenfrequency can be effectively tuned by the DC voltage applied to the tuning electrode to achieve mode-matching operation. Lumped mass design is used to reduce the initial frequency split. The piezoelectric electrodes are designed according to the stress pattern of operating modes, and the electrical decoupling of IP and OOP modes is achieved through vectors operation to reduce cross-coupling.

Experimental results demonstrate that the gyroscope achieves mode-matching through electrostatic tuning, and a frequency split of 0.1 Hz is obtained without the need for any mechanical trimming. The sensitivity improvement after mode-matching meets theoretical expectations, and both the angle random walk (ARW) and bias instability (BI) have been significantly enhanced. The results of ADEV show that the BI of the presented gyroscope is comparable to that of the highest-performing mechanically trimmed yaw piezo-MEMS gyroscope known to date^14^, validating the effectiveness and significant potential of our design.

## Results and discussion

### Mode-shape design

For a rate gyroscope, assume the drive mode amplitude to be *q*_*d*_, the mechanical sensitivity of a rate gyroscope can be written as:

1-1 $${S}_{{mech}}=\frac{2\lambda {q}_{d}}{\sqrt{{\left(\frac{{{\omega }_{s}}^{2}}{{\omega }_{d}}-{\omega }_{d}\right)}^{2}+\frac{{{\omega }_{s}}^{2}}{{{Q}_{s}}^{2}}}}$$

*Qs* is the quality factor of sense mode, ${\omega }_{s}$,${\omega }_{d}$ are angular frequency of sense and drive mode. $\lambda$ representing the Coriolis coupling coefficient, which is defined as the ratio of the Coriolis mass to the equivalent mass of the sense mode. For an x-axis(roll-axis) gyroscope, the $\lambda$ can be given as:

1-2 $$\lambda =\frac{{m}_{c}}{{m}_{2}}=\frac{\int ({U}_{1}\times \mathop{\varOmega }\limits^{\frown {}})\ast {U}_{2}dV}{\int {U}_{2}^{2}dV}=\frac{\int ({U}_{y1}{U}_{z2}-{U}_{z1}{U}_{y2})dV}{\int {U}_{2}^{2}dV}$$

where $\mathop{\varOmega }\limits^{\frown {}}$ is the unit vector of rotation, *U*_*1,2*_ are mode-shape functions for the normalized displacement field, *Uy*_*1,2*_ and *Uz*_*1,2*_ the y component of normalized displacement field, and the z component of normalized displacement field of the drive and sense modes, respectively.

When the frequency of the drive and sense modes is matched, ${\omega }_{d}$ is equal to ${\omega }_{s}$, the above equation can be simplified as:

1-3 $${S}_{mech}=\frac{2\lambda \mathop{\varOmega }\limits^{\frown {}}{Q}_{s}}{{\omega }_{s}}{q}_{d}$$

Equation (1–3) can also be written as relationship between driving amplitude *q*_*d*_ and sensing amplitude *q*_*s*_ actuated by Coriolis force:

1-4 $${q}_{s}=\frac{2\lambda \mathop{\varOmega }\limits^{\frown {}}{Q}_{s}}{{\omega }_{s}}{q}_{d}$$

Mechanical noise equivalent rate output can be given by:

1-5 $${MNE}\varOmega =\frac{1}{2\lambda {q}_{d}}\sqrt{\frac{4{k}_{B}T}{{\omega }_{0}{M}_{S}{Q}_{S}}}$$

**Fig. 1:**
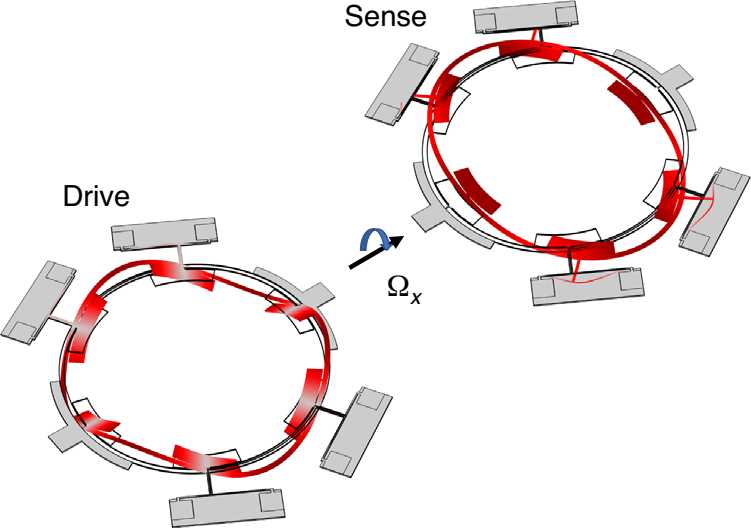
Mode shape of operation modes. OOP mode(left) is driven and generate IP mode(right) vibration when roll axis rotation is applied

It can be seen that the sensitivity and signal-to-noise ratio of the gyroscope are highly reliant on the $\lambda$ and *q*_*d*_. The presented annulus solid-wave gyroscope uses *n* = 2 IP wineglass mode and *n* = 3 OOP bending mode as the operation modes which are illustrated in Fig. 1. To achieve sensitivity along the x-axis(roll), the device is designed to utilize the OOP mode as drive mode. When an x-axis rotation is applied, it causes IP coupling and generates a sense current. $\lambda$ is found to be around 0.15 through FEM, which is acceptable for gyroscopic operation^22–24^.

#### Mechanical mode-matching design

**Fig. 2:**
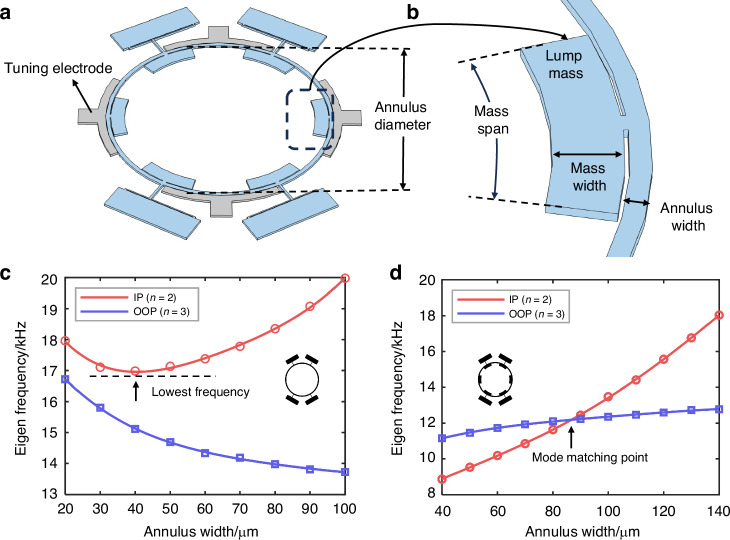
Schematic and simulation results of mechanical mode-matching design. **a** Main structure schematic of the presented gyroscope **b** Structure of lump mass **c** Eigen frequencies variation in the IP mode and OOP mode of the conventional annulus (without lump mass) **d** After applying lumped masses, the frequencies of the IP and OOP modes with varying annulus

It is necessary to design the eigenfrequencies of the gyroscopic modes close to each other using Finite Element Method (FEM) tools to achieve mechanical mode matching. Figure 2c shows the simulated changes in eigenfrequencies in the IP mode and OOP mode of the conventional annulus as the annulus width decreases from 100 μm to 20 μm. The device has a thickness of 25 μm and an outer diameter of 5000 μm. The annulus is suspended by four T-shaped anchors located at nodes of the OOP mode. As the annulus width decreases from 100 μm to 40 μm, the eigenfrequency of the *n* = 2 IP mode decreases, while the eigen frequency of the *n* = 3 OOP mode increases. This is mainly due to the contribution of stiffness in the width direction to the IP mode being greater than OOP mode. However, due to the influence of anchor stiffness, the eigenfrequency of the IP mode is not monotonically decrease when the annulus width is reduced. An inflection point is observed in Fig. 2c, and further decreasing the annulus width to less than 20 μm will lead to a decrease in the piezoelectric transduction efficiency and make fabrication difficult.

Therefore, a stiffness-mass decoupling design was applied, as shown in Fig. 2a. The method of lumping mass blocks is often used in multi-ring gyroscope designs to increase the vibrational mass^3^. But in our design, the lumped mass blocks are primarily used to match the eigenfrequencies of IP and OOP modes. The mass shape is designed as a fan connected to the annulus by a small stick, as shown in Fig. 2b. Masses are placed at the nodes of the OOP mode. After applying lumped masses, the frequencies of the IP and OOP modes with varying annulus are shown in Fig. 2d. It can be observed that the frequencies of the two modes match at a width of 90 μm.

Ultimately, an annulus with an outer diameter of 5000 μm, a width of 90 μm, and a device layer thickness of 25 μm is selected, with the dimensions of the lumped mass shown in Fig. 2(b).

### Electrostatic mode-matching design

Electrostatic tuning can mitigate mode split in gyroscopes caused by photolithography errors or crystal imperfections after fabrication. Electrostatic mode-matching design utilizing the spring softening effect, which comes from the electrostatic force. Electrostatic stiffness of a gap-closing parallel plate capacitive transducer can be described in Eq. (2–1)

2-1 $${k}_{{electrostatic}}=-{{V}_{P}}^{2}{{\cdot }}\frac{{C}_{0}}{{g}^{2}}=-{{V}_{P}}^{2}{{\cdot }}\frac{\varepsilon\, {{\cdot }}\,A}{{g}^{3}}$$

Where *Vp*, *g*, *C*_*0*_, *ε* are the polarization voltage across the parallel plate, the gap size, initial capacitance value and the dielectric constant, respectively.

The pull-in voltage V_PI_ and pull-in gap size *g*_*PI*_ can be written as:

2-2 $${V}_{{PI}}=\sqrt{\frac{8k{{g}_{0}}^{3}}{27\varepsilon A}}$$

2-3 $${g}_{{PI}}=\frac{2}{3}{g}_{0}$$

**Fig. 3:**
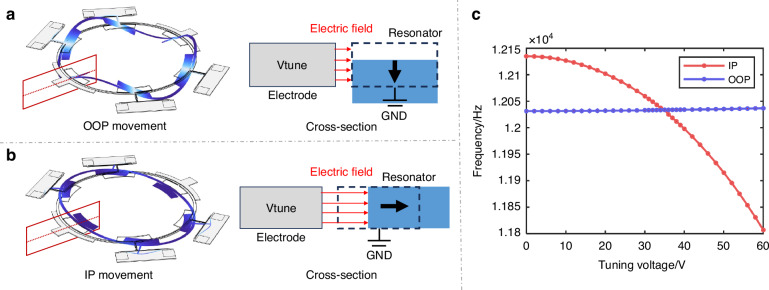
Schematic and simulation results of electrostatic mode-matching design. **a** Tuning electrodes and movement of OOP mode, neither significant electrostatic negative stiffness nor displacement limitation will be caused by electrostatic gap**. b** Tuning electrodes and movement of IP mode. It can be regard as a parallel plate. **c** The eigen frequency simulation results of IP and OOP as the tuning voltage increasing

Where *g*_*0*_ is the initial gap size of parallel plate. From Equation (2–1), it is known that a smaller gap size can reduce the required voltage *Vp* for generating the same electrical stiffness, which is beneficial for mode-matching operations. However, when the displacement *q*_*d*_ in Equation (1–4) exceeds 1/3 g_0_, it can cause pull-in, leading to a short circuit and damage the device. Therefore, there is a trade-off between electrostatic tuning efficiency and driving amplitude. The trade-off is released in our design by implementing electrostatic tuning electrodes only in the sense mode. According to Equation (1–4), the amplitude of the sense mode actuated by the Coriolis force is usually significantly smaller than that of the drive mode, resulting in less impact of the electrostatic gap size. Moreover, since the OOP and IP movements are orthogonal, as shown in Fig. 3a, b, the OOP amplitude is not significantly limited by the IP mode’s parallel plates. Even if the amplitude of OOP is large, the minimum distance between the resonator and the electrode will not change significantly, especially when the parallel plate is positioned at the nodes of the OOP (*n* = 3) mode.

Therefore, four tuning electrodes are implemented at the antinodes of the IP (*n* = 2) mode, as illustrated in Fig. 2a. Applying a tuning voltage to these electrodes effectively lowers the IP mode’s eigenfrequency, while leaving the OOP mode’s eigenfrequency almost untuned. Therefore, the untuned IP frequency is intentionally designed to be slightly higher than the OOP mode to ensure mode-matching capability. Ultimately, the IP mode frequency was designed to be 12.13 kHz, and the OOP mode frequency to be 12.03 kHz, with the width of the electrostatic gap set at 4 μm. The effectiveness of the tuning was verified through FEM simulations in COMSOL Multiphysics. As illustrated in the Fig. 3c, as the DC tuning voltage is gradually increased, the IP frequency exhibits a quadratic decrease, and the modes are matched at 36 V. This simulation results validate the effectiveness of tuning design.

### Piezoelectric electrodes and decoupling vectors operation

**Fig. 4:**
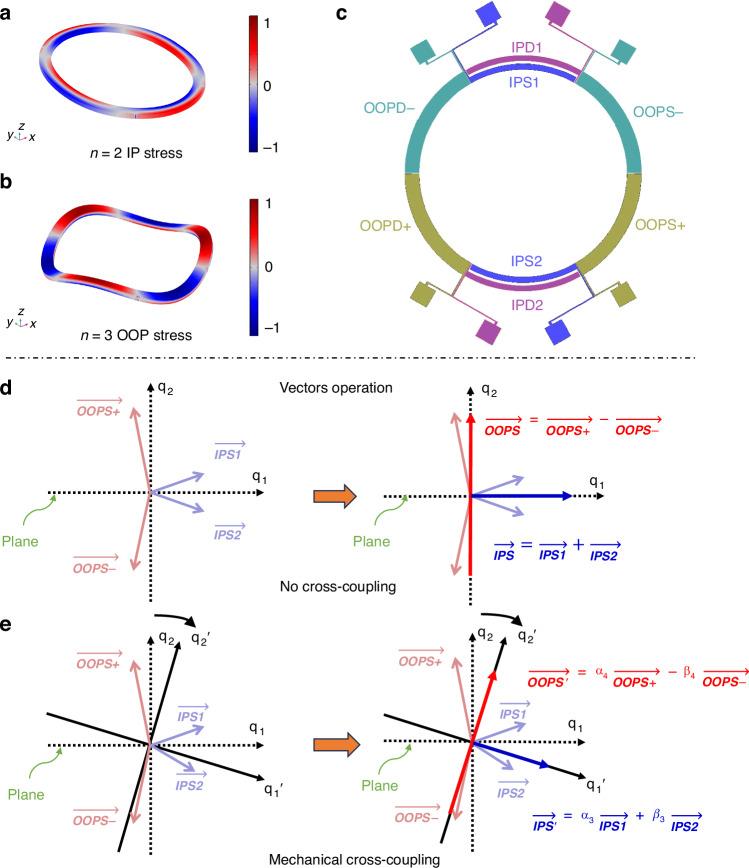
Piezo electrodes pattern and schematic of vector operation. **a** IP mode (*n* = 2) stress pattern **b** OOP mode (*n* = 3) stress pattern **c** Piezoelectric electrodes pattern **d** Schematic of ideal vectors operation without mechanical cross-coupling. Vectors can be aligned by differential and summation operation. **e** Vectors operation considered mechanical cross-coupling. The mode coordinate {q’}is orthogonal and has an angular offset from the general coordinate {q}. coefficients α and β are needed to tune the gain of the vector basis so that computed vectors can be aligned with coordinate {q’}

Since the input/output signal of a piezoelectric resonator represents stress instead of displacement, the electrodes configuration is designed according to the stress pattern. The stress pattern for the IP mode is illustrated in Fig. 4a. The annulus exhibits in-plane bending, generating a stress gradient across its width, with opposite stress directions on either side of the neutral axis. In contrast, the OOP mode involves bending and twisting, creating a stress gradient oriented along the thickness of the annulus. Piezoelectric electrode patterns are illustrated in Fig. 4c. Four OOP electrodes and four IP electrodes are positioned at the nodes of the OOP mode and IP mode to enhance the electromechanical coupling coefficient in the desired vibration direction. However, a single electrode can still actuate or capture vibrations of another undesired operational mode due to mechanical and electrical stiffness coupling, which can bring long-term noise and increase the minimum frequency split of mode matching operation. Therefore, a decoupling design called vectors operation is proposed to achieve alignment of electrical and mechanical coordinate axes.

The principle of vectors operation is based on eigenmode operation, which has been explained in detail in previous works^6,14^ and has been successfully used in yaw piezoelectric gyroscopes. It has been adapted for the first time to pitch/roll piezoelectric gyroscopes in this work. The schematic of vectors operation is shown in Fig. 4d. Four vectors OOPS + , OOPS-, IPS1, and IPS2 in the left coordinate of Fig. 4d represent the sense currents of the electrodes with the same names in Fig. 4c, respectively. The coordinate axes of dashed lines represent mechanical vibrations in the OOP(q_1_) and IP(q_2_) directions. The amplitude and phase of each vector represent the stress pattern in Fig. 4a, b. First, we assume that the shape of the electrodes is perfectly symmetrical and that there is no mechanical stiffness coupling. It is obvious that sensing currents on the OOPS+ and OOPS- share the same phase on IP direction, while the vectors of IPS1 and IPS2 share opposite phases in the OOP direction. Therefore, differential currents of OOPS+ and OOPS- can suppress the detection of IP vibrations in the OOP channel. In the same way, the summation currents of IPS1 and IPS2 suppress OOP vibration in the IP channel. Due to the symmetry of the electrode design, the driving and sensing processes are opposite. Applying in-phase signals on IPD1 and IPD2 can drive the IP mode and suppress the vibration of the OOP mode. Similarly, applying differential signals to OOPS+ and OOPS- can effectively operate the OOP mode without inducing vibration in the IP mode. When considering mechanical coupling, slight adjustments need to be made to the vectors operation described above. Due to cross-coupling, the mode coordinate, which is orthogonal, has an angular offset from generalized coordinate, as shown by the solid black line in Fig. 4e. Therefore, coefficients α and β are needed to tune the gain of the vector basis, aligning the computed vectors with the mode coordinate to achieve decoupling. Figure 4e illustrates this process on the sensing side with α_3,4_ and β_3,4_, and the same procedure applies to the driving side with α_1,2_ and β_1,2_.

In short, vectors operation takes signals on the electrodes as the basis vectors and construct electric vector axes aligned with the mechanical coordinate, thereby achieving cross decoupling.

### Fabrication

**Fig. 5:**
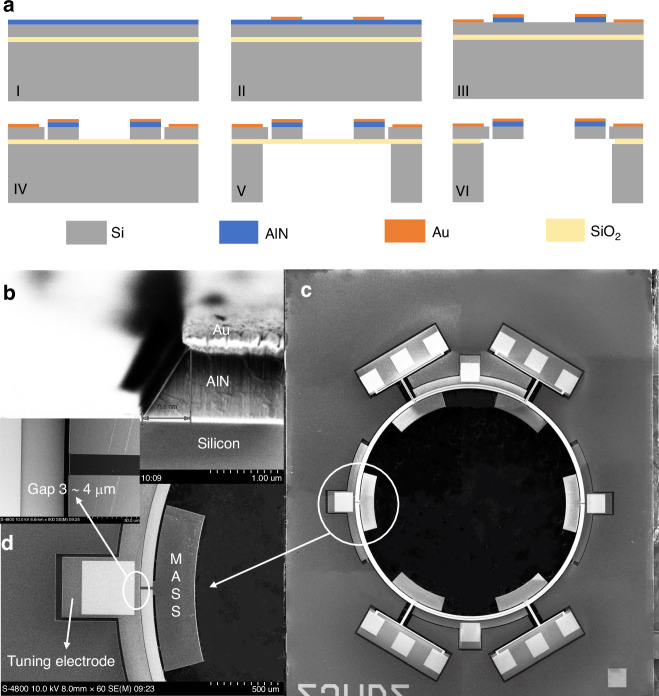
Fabrication process of the thin piezoelectric film on silicon gyroscope and SEM pictures of fabricated device. **a** Four-mask fabrication process. The top electrode layer is used as a mask to pattern the AlN using a 25% TMAH solution at room temperature. **b** SEM pictures of layer stack. **c** Fabricated gyroscope. **d** A close-up view of the lumped mass and the electrostatic tuning electrodes

The cross-sectional view of the four-mask fabrication process of the piezoelectric gyroscope is shown in Fig. 5a. The device utilizes a TPoS structure on a SOI substrate, featuring a 1-micron thick AlN film and a 25-micron thick device layer. The SOI device layer is highly doped, allowing the piezoelectric material to be directly grown on silicon without the need for a bottom metal electrode.

Initially, the piezoelectric layer is deposited on the highly doped silicon substrate. After that, the top Au electrode layer is sputtered and patterned to be the top signal electrodes. The top electrode layer is used as a mask to pattern the AlN using a 25% TMAH solution at room temperature^25–27^. Subsequently, Au electrodes are sputtered and patterned on silicon as pads for wire bonding. The device layer is etched to pattern the device structure and electrostatic gaps. Finally, the handle layer and buried oxide layer are etched from backside to release the device^28^.

SEM pictures of electrodes and piezo and silicon layers stack are shown in Fig. 5b. A fabricated gyroscope is shown in Fig. 5c. Figure 5d presents a close-up view of the lumped mass and the electrostatic tuning electrodes, and an electrode gap of approximately 4 μm. Key parameters of the presented gyroscope are shown in Table 1.

**Table 1:** The Key parameters of gyroscope

Parameter	Value
AlN thickness	1[μm]
Silicon device layer thickness	25[μm]
Annulus diameter	5000[μm]
Annulus width	90[μm]
Mass span	30[deg]
Mass width	320[μm]
Electrostatic gap	4[μm]
Tuning electrode angle	58[deg]
Support beam width	20[μm]

### Experimental results

**Fig. 6:**
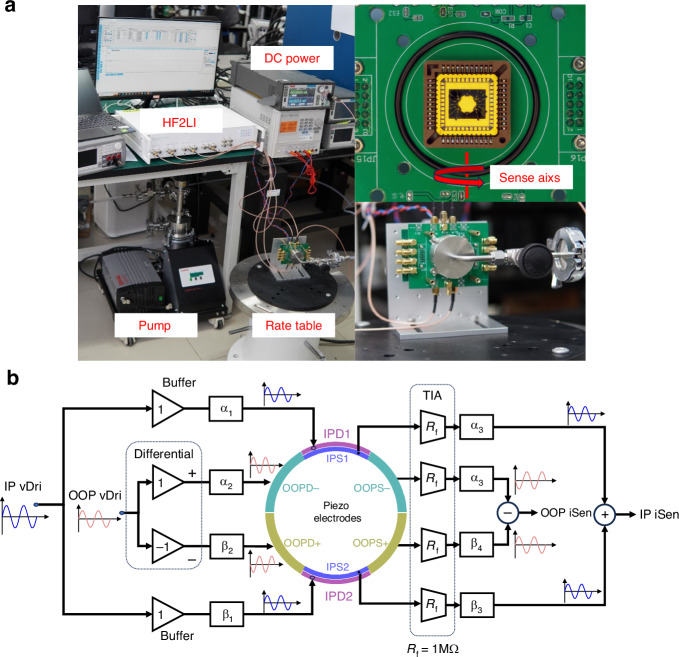
Mechanical and electrical test setup. **a** Test environment and installation of gyroscope on a single axis rate table. Gyro was performed in vacuum. **b** Schematic of analog PCB and vectors operation circuit. Multi-coefficients of α and β can be changed using a sliding rheostat on board

The experimental measurement was performed in a customized vacuum chamber using mechanical and molecular pumps and a sealed cap placed on top of printed circuit boards(PCB) as shown in Fig. 6(a). The vacuum gauge indicated that the vacuum level within the chamber could reach 0.01 Pa, allowing for negligible squeeze-film damping (SFD). The gyroscope is wire-bonded to an Leadless-Ceramic-Chip-Carrier(LCC44) socket on an analog PCB containing front circuits of vector operations. Analog PCB is connected to a Zurich Instruments HF2LI Lock-in Amplifier via SMA interfaces. The under-test gyroscope is fixed vertically on a single-axis rate table using screws so that in-plane rotation can be applied to the roll axis of gyroscope.

#### Resonator response

Sweep results were conducted to characterize the resonator behavior of the gyroscope. At room temperature, the quality factors (Q) for the IP and OOP modes were 10,345 and 8125, respectively. Vectors operation with multi-coefficients is applied as shown in Fig. 6b. The in-phase IP driving signals are applied through a pair of buffers and the out-of-phase OOP driving signals are applied through a pair of differential buffers. Similarly, the currents of sensing electrodes are amplified by 4 TIAs and the common mode signal which represents IP mode vibration is extracted using an analog adder while the differential signal which represents OOP mode vibration is extracted using an analog subtractor. The amplitude of driving or sensing on each electrode can be tuned by a coefficient α and β, which are set using sliding rheostats on board.

**Fig. 7:**
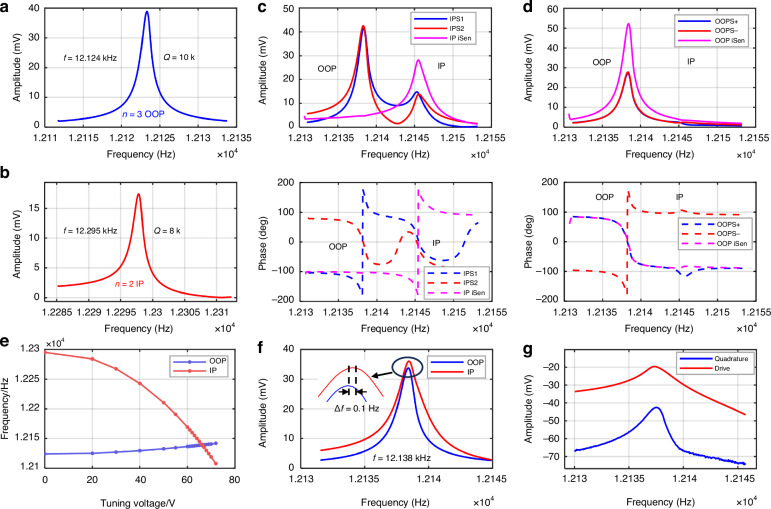
Mode-matching and vectors operation results. **a** Untuned OOP mode peak. **b** Untuned IP mode peak. **c** Vectors operation of IP mode, OOP peaks(out-of-phase) are suppressed while IP peaks(in-phase) are doubled after an analog adder. **d** Vectors operation of OOP mode, IP peaks(in-phase) are suppressed while OOP peaks(out-of-phase) are doubled after an analog subtractor. **e** As the tuning voltage increases, the eigen frequency test results of IP and OOP mode. Modes matched at a Vp of 66.35 V.**f** A minimum frequency split of 0.1 Hz is obtained after vectors operation at Vp of 66.35 V. **g** Quadrature response shows a 23 dB cross-mode isolation of mode matched

The as-fabricated frequency mismatch is 171 Hz at an initial tuning voltage Vp of 0 V. As Vp increasing, the mode-split becomes smaller. Simultaneously sweep the IP and OOP mode and record TIAs outputs and sense currents after vector operations. Figure 7c exhibited the currents of IPS1 and IPS2 electrodes and the output of the analog adder. Although the currents of each TIA captured peaks of OOP mode, they cancelled each other out after multiplying by the coefficients and passing the adder. Similarly, Fig. 7d shows the current of the OOPS +and OOPS- electrode and the output of the subtractor.

By repeated fine-tuning of Vp and coefficients α and β, the two modes matched at approximately 12.14 kHz and a minimized frequency split of 0.1 Hz is obtained as Fig. 7e, f shown, validating the effectiveness of mode-matching design. Figure 7g exhibits the quadrature response of the OOP channel while sweeping IP mode when the modes are almost matched. The cross-mode isolation after vector operations is approximately 23 dB, which is within the normal range according to previous work^14^.

#### Gyroscope performance

Gyroscope performance of the device is characterized with an HF2LI for phase-locked loop (PLL) and automatic gain control (AGC) in the OOP driving loop. The gyroscope is manually mode-matched as described above, and the tuning voltages are maintained constant when the optimized tuning condition has been established.

The actuation voltage applied to OOP+ and OOP - is 47 mV, and an OOP antinode driving amplitude of 3 μm can be derived by detecting the vibrating voltage in the readout circuit of the driving loop, which is larger than the max displacement allowed by electrostatic driving^3,21–23^. The sensing currents of IP mode is demodulated in the lock-in amplifier to obtain the open loop rate reading.

**Fig. 8:**
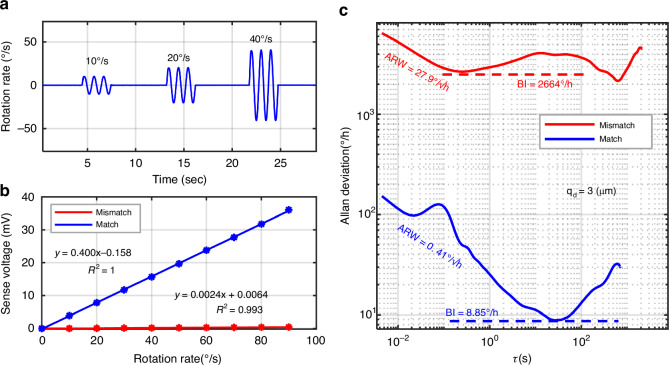
Gyroscope performance. **a** Transient gyroscope sensing output response to rotations. **b** The measured sensitivity of match and mismatch mode. **c** ADEV results of the gyro of match and mismatch mode

The time-domain output waveform of the gyroscope is shown in Fig. 8a over a period of 50 s. The input mechanical rotation rate to the device is 10, 20, 40°/s, applied at a frequency of 1 Hz. Rate response up to 90°/s is measured at the frequency of 0.25 Hz, where the applicable rotation rate is limited by the testing setup. The presented gyroscope shows a mode-match voltage sensitivity of 400 μV/(°/s) and a sensitivity of 24 μV/(°/s) in the mode-mismatch which are shown in Fig. 8b. Under the same driving amplitude *q*_*d*_ of 3 μm, mode matching operation exhibited as high as 167x improvement, which is close to the theory employs equation (1–1) of 200x. The error between theoretical and experiment is possibly caused by the phase modulator and non-ideal manufacturing. The cross-axis sensitivity is measured by manually placing the PCB in different orientations, showing cross-axis sensitivity of 0.88% for pitch rotation and 0.25% for yaw rotation, demonstrating good cross-axis suppression.

ARW and BI are tested at room temperature. The measured ADEV (Fig. 8(c)) shows that the gyroscope has an open loop ARW of 0.41°/√h and BI of 8.85°/h under mode matching operations, illustrating a 68x and 301x improvement compared to that of mode-mismatch condition with 171 Hz mode split at a same driving amplitude. The theoretical Brownian noise ARW of the gyroscope can be calculated as low as 0.06°/√h, indicating the main source of short-term noise is circuit noise. The bias instability level occurs when the time constant τ is greater than 10 s, indicating a rejection of the quadrature error. The long-term noise observed after 100 s is primarily due to the vibrations from pumps. The poor performance of BI of mode-mismatched is primarily due to an increase in mechanical and electrical noise, which directly impacts the stability and accuracy of the gyro.

Table 2 gives a summary of the comparison with previously reported state-of-the-art piezo-MEMS gyroscopes and electrostatic pitch or roll gyroscopes in academia and industry. As can be seen from Table 2, the open loop BI performance of proposed pitch or roll gyroscope is comparable to that of the highest-performing mechanically trimmed AlN-on-Si yaw gyroscopes as known^14^, while offering advantage of mode-matching resolution and the flexibility of real-time mode-matching operation, showing a great potential to achieve better precision. Moreover, the performance of this gyroscope is also close to that of commercial yaw piezo-MEMS gyroscopes of Silicon sensing^29^, even though the latter have advantages of a larger piezoelectric coefficient and a lower noise ASIC circuit. It is also illustrated that BI and ARW of piezoelectric gyroscope could be on par with that of electrostatic mode-matched pitch/roll gyroscopes^30^.

**Table 2:** Comparison of State-of-the-Art Piezo-MEMS and Pitch or Roll Gyroscope Performance

Parameters	This work	GA Tech^14^	Silicon Sensing^29^^a^	GA Tech^30^
Year	2024	2023	2015	2020
Gyro.Type	Pitch/roll	Yaw	Yaw	Pitch/roll
Operation Mode	Matched	Matched	–	Matched
Tuning method	Electrical	Mechanical	None	Electrical
Transduction	Pizeo(AlN)	Pizeo(AlN)	Pizeo(PZT)	Electrostatic
ARW(°/√h)	0.41	0.145	0.2	0.45
BI(°/h)	8.85	8.6	7.2	12.8

^a^BI is acquired from ADEV curve in datasheet

## Discussion and conclusion

In this paper, we introduced a piezo-MEMS pitch or roll gyroscope with BI below 10°/h. To our knowledge, this is the first time that electrostatic mode-matching design has been achieved on a piezoelectric pitch or roll gyroscope. The design of the sensing mode electrostatic electrode in the IP direction enables compatibility between electrostatic mode-matching and the large driving amplitude in the OOP direction. The vectors operation of piezoelectric electrodes achieves electrical decoupling, and mode matching operation improves the noise performance of the gyroscope. For future work, the gyroscope structure will be enhanced to improve its Coriolis coupling coefficient and signal-to-noise ratio. Device or wafer-level packaging has been considered to eliminate the need for external vacuum pumps. Interface circuits with real-time frequency control will also be further explored to enhance BI performance.
